# Xenophobia and intercultural sensitivity among nursing and midwifery students: a cross-sectional study

**DOI:** 10.1186/s12912-026-04868-6

**Published:** 2026-06-26

**Authors:** Beril Nisa Yaşar, Gülhan Erkuş Küçükkelepçe

**Affiliations:** 1 Present Address: Department of Midwifery, Faculty of Health Sciences, Mardin Artuklu University, Mardin, Türkiye; 2 Present Address: Department of Nursing, Faculty of Health Sciences, Adıyaman University, Adıyaman, Türkiye

**Keywords:** Intercultural sensitivity, Xenophobia, Nursing students, Midwifery students, Cultural competence, Nursing education

## Abstract

**Background:**

As healthcare settings become more culturally diverse, nursing and midwifery education needs to prepare students to provide culturally responsive care. Although intercultural sensitivity has received growing attention in health professions education, less is known about how it relates to negative attitudes such as xenophobia. This study examined levels of xenophobia and intercultural sensitivity among nursing and midwifery students and explored their relationships with selected sociodemographic and experiential characteristics.

**Methods:**

This descriptive cross-sectional study was conducted with 472 nursing and midwifery students enrolled at a state university in Mardin, Türkiye. Data were collected using a sociodemographic questionnaire, the Xenophobia Scale, and the Intercultural Sensitivity Scale. The data were analyzed using descriptive statistics, correlation analysis, and multiple regression analysis.

**Results:**

The students had moderate xenophobia scores and relatively high intercultural sensitivity scores, with mean values of 51.83 ± 12.74 and 88.25 ± 12.63, respectively. No significant relationship was found between total xenophobia and total intercultural sensitivity scores. Weak positive correlations were found between xenophobia and the interaction responsibility (*r* = 0.105, *p* = 0.023) and interaction carefulness (*r* = 0.243, *p* < 0.001) subdimensions of intercultural sensitivity. Xenophobia scores differed significantly by age, academic year, geographic region, and frequency of intercultural interaction, although the effect sizes were small (η² = 0.02–0.04). In the regression analysis, desire to live abroad remained a significant predictor of higher xenophobia scores (B = 5.834, *p* < 0.001).

**Conclusions:**

Xenophobia and intercultural sensitivity do not appear to move together in a straightforward way among nursing and midwifery students. The findings suggest that being sensitive to cultural differences does not necessarily mean being free from xenophobic attitudes. Nursing and midwifery education may therefore benefit from addressing both aspects to better support the development of culturally responsive care.

## Introduction

Contemporary global challenges, including escalating globalization, natural disasters, unemployment, and migration, compel individuals to seek improved living conditions, resulting in substantial global migration and increased intercultural contact. This evolving demographic landscape necessitates adaptations in healthcare service demands and delivery, fostering new concepts and approaches. The growing cultural and ethnic diversity of societies underscores the critical need for culturally congruent healthcare, reaffirming the importance of culturally sensitive access [1]. While heightened cultural diversity emphasizes personalized and culturally sensitive care, it also presents novel complexities in healthcare planning and delivery. Effective healthcare provision necessitates that health professionals respect patients’ cultural identities and values, integrating these differences into their practice. Globalization and migration-induced multiculturalism have fundamentally altered healthcare delivery. Alongside advancements like culture-specific care, negative attitudes such as xenophobia and ethnocentrism have emerged. Failure of healthcare systems to adapt may contribute to increased inequalities in access and poorer health outcomes, underscoring the importance of culturally responsive health policies and cultural sensitivity training for healthcare professionals [2].

Xenophobia, defined as negative attitudes and behaviors towards different cultures [3], can deprive individuals of fundamental human rights and exacerbate social inequities. Effective intercultural healthcare demands cultural sensitivity, characterized by understanding and respecting diverse cultural values and beliefs. Healthcare staff’s cultural sensitivity is considered important, particularly in multicultural contexts, as it has been associated with reduced discrimination and inequality, greater patient and provider satisfaction, and better health outcomes [4].

Although xenophobia and intercultural sensitivity are often conceptualized as opposing constructs, their relationship among healthcare students appears to be more complex. Xenophobia reflects fear-based negative attitudes toward cultural outgroups [5], whereas intercultural sensitivity represents an affective openness toward cultural diversity [6]. While previous studies have generally reported an inverse association between these constructs [7, 8], emerging evidence suggests that they may coexist under certain conditions [9]. In addition, contextual, educational, and sociocultural factors may shape this relationship. For example, negative intercultural experiences, perceived economic or security threats, and migration-related social discourse may contribute to xenophobic attitudes, whereas educational and clinical experiences focused on culturally responsive care may support intercultural sensitivity [10]. These findings suggest that xenophobia and intercultural sensitivity may not function as strictly opposite dimensions, highlighting the importance of examining both constructs simultaneously.

Despite growing interest in intercultural sensitivity and xenophobia in health-related student populations, empirical evidence exploring how these two constructs coexist and interact remains limited. In particular, little is known about the sociodemographic and educational factors associated with both xenophobic attitudes and intercultural sensitivity among nursing and midwifery students who will provide care in culturally diverse healthcare environments.

This study was guided by complementary theoretical perspectives addressing both positive and negative orientations toward cultural diversity. Intercultural sensitivity was conceptualized as the affective dimension of intercultural communication competence [6], whereas xenophobia was defined as a fear-based attitudinal response toward cultural outgroups [5]. These constructs were considered individual-level attitudes influencing interactions with culturally diverse patients. In addition, elements of Ecological Systems Theory were considered to account for the influence of sociodemographic and educational contexts. This perspective is consistent with prior research examining xenophobia among nursing students [10].

At the individual level, xenophobic attitudes may also have important implications for nursing and midwifery students themselves. Such attitudes may hinder the development of effective professional relationships with culturally diverse peers and patients, reduce willingness to engage in intercultural communication, and limit opportunities for cultural learning and professional growth [4, 10]. In addition, xenophobic tendencies may conflict with the principles of culturally responsive and patient-centered care that constitute core professional values in nursing and midwifery practice [11]. Students with higher xenophobic attitudes may therefore experience difficulties in developing the cultural competence required for effective practice in multicultural healthcare environments [12, 13].

This issue also has practical implications for patient care and equity. Xenophobia-related attitudes may be reflected in exclusionary orientations or behaviors that can hinder migrant-sensitive and equitable care [2, 11]. In contrast, intercultural competence and the quality of intercultural contact have been linked to lower xenophobia, pointing to the value of educational approaches that strengthen competence and support meaningful contact [14]. Examining xenophobia and intercultural sensitivity together therefore offers a more informative perspective on students’ preparedness for culturally competent care.

Developing intercultural competence in future nurses and midwives has become a central objective of contemporary nursing education. Undergraduate programs are expected to prepare students not only with clinical knowledge but also with the attitudes and communication skills required for culturally responsive care. Understanding how xenophobia coexists with intercultural sensitivity is therefore essential for designing effective educational interventions. Evidence suggests that various sociodemographic and experiential factors may influence these attitudes.

The increasing globalization of healthcare requires intercultural competence among healthcare professionals, as they frequently provide care to culturally diverse populations. Given the growing significance of nursing and midwifery in diverse healthcare settings, re-evaluating education to foster intercultural sensitivity and address xenophobic attitudes is essential for the delivery of high-quality, inclusive care. Therefore, this study aimed to examine xenophobia and intercultural sensitivity among nursing and midwifery students, to explore the relationship between these constructs, and to identify associated sociodemographic and experiential factors. By examining xenophobia and intercultural sensitivity together rather than in isolation, this study seeks to provide a more comprehensive understanding of the attitudinal dimensions that may influence culturally responsive nursing and midwifery practice. Simultaneously evaluating both negative attitudes toward cultural outgroups and positive orientations toward cultural diversity may help identify educational needs beyond knowledge and communication skills alone. This combined perspective may contribute to the development of educational strategies aimed at strengthening cultural competence, reflective awareness, and equitable care practices among future healthcare professionals.

*Study questions*.

1. What are the xenophobia and intercultural sensitivity levels of nursing and midwifery students?
2. Do xenophobia and intercultural sensitivity levels of nursing and midwifery students differ according to selected sociodemographic and experiential characteristics, such as age, sex, grade level, geographic background, and intercultural contact experiences?
3. Is there any relationship between xenophobia and intercultural sensitivity levels of nursing and midwifery students?

## Methods

### Study design

The study employed a descriptive, cross-sectional survey design.

### Participants, setting, and recruitment

The study was conducted at Mardin Artuklu University, Faculty of Health Sciences, in Mardin, Türkiye, between May and June 2023. Mardin is located in the Southeastern Anatolia Region and is adjacent to the Syrian border; therefore, cultural diversity and cross-cultural contact constitute a relevant part of the local context. During the study period, Türkiye hosted a large refugee population, including approximately 3.6 million Syrians under temporary protection and displaced populations from other countries [15].

At the time of the study, the nursing and midwifery programs at Mardin Artuklu University did not include a standalone course specifically dedicated to intercultural nursing or transcultural care. However, topics related to cultural diversity, refugee health, communication with culturally diverse patients, and culturally sensitive care were addressed within the scope of existing courses such as Public Health Nursing, Transcultural Midwifery, and clinical practice courses. In addition, students completed their clinical placements in healthcare institutions within Mardin province, where they were likely to encounter patients from diverse cultural and linguistic backgrounds, including Syrian refugees and individuals from different ethnic communities. The multicultural structure of the region and the proximity to the Syrian border provide students with frequent opportunities for intercultural interaction in both social and clinical settings.

The study population comprised all students actively enrolled in the nursing and midwifery programs during the 2022–2023 academic year (*N* = 729). International (foreign national) students (*n* = 92) were excluded to ensure alignment with the intended target group of the Xenophobia Scale, which assesses host-population attitudes toward immigrants/outgroups; thus, the eligible population consisted of 637 students. Participation was voluntary, and 472 students completed the survey, corresponding to a participation rate of 74.09%.

Recruitment was carried out by disseminating the online survey link through WhatsApp class-group announcements and the official social media accounts of the relevant faculty (access limited to students in the department). To minimize any potential perceived pressure to participate, announcements were shared via class representative students rather than teaching staff. The first page of the online survey presented study information and an electronic consent statement; only students who provided consent proceeded to the questionnaire, and participants were informed that they could withdraw at any time by exiting the survey without any consequences.

### Data collection tools

The data were collected online by using the Introductory Information Form, Xenophobia Scale, and Intercultural Sensitivity Scale.

### Introductory information form

The form was prepared by the researchers in line with the literature and consists of 13 questions covering the demographic characteristics of the participants (age, sex, etc.), their cultural interaction experiences (experience abroad, frequency of interaction with different cultures), and their cultural awareness in healthcare services (experience in providing care to patients from different cultures).

### Xenophobia scale

Developed by Veer, Ommundsen, Yakushko, and Higler [5] and adapted to Turkish by Özmete, Yıldırım, and Duru [16], the scale is one-dimensional and consists of 11 items. In the Turkish adaptation study, the scale was organized as a single factor. In the present study, the unidimensional structure reported in the Turkish adaptation study was adopted, and analyses were conducted using the total scale score. The high internal consistency coefficient obtained in the current sample (Cronbach’s α = 0.924) also supported the use of the scale as a single-factor measure in this study population. The Xenophobia Scale is a 6-point Likert-style scale and the items are answered as “I Strongly Disagree (1)” to “I Strongly Agree (6)”. The scale is one-dimensional and item 8 is reverse-coded. The lowest score that can be obtained from the scale is 11, and the highest score is 66. The scale conceptualizes xenophobia as interpersonal attitudes toward immigrants and refugees, measuring fear-based negative attitudes. An increase in the total score obtained from the scale indicates an increase in the risk of xenophobia. The Cronbach Alpha value of the scale was found to be 0.87. In the present study, it was found to be 0.924.

### Intercultural sensitivity scale

The scale was developed by Chen and Starosta [6] and adapted into Turkish by Bulduk, Tosun, and Ardıç [17]. It consists of five sub-dimensions and 24 items. The Intercultural Sensitivity Scale is a five-point Likert-type scale, and the items are answered as “5 = I Strongly Agree, 4 = I Agree, 3 = I am Undecided, 2 = I Disagree, and 1 = I Strongly Disagree”. The responsibility in interaction dimension of the scale (items 1, 11, 13, 21, 22, 23, 24) assesses feelings related to participation in intercultural communication and proactive tendency to engage with diverse others. The respect for cultural differences dimension (items 2, 7, 8, 16, 18, 20) assesses approaches to different cultural orientations and appreciation of diverse values and beliefs. The self-confidence in interaction dimension (items 3, 4, 5, 6, 10) assesses the level of self-confidence in an environment with intercultural differences. The interaction enjoyment sub-dimension (items 9, 12, and 15) assesses positive or negative reactions to communicating with people with cultural differences. The interaction attentiveness sub-dimension (items 14, 17, and 19) assesses efforts to understand what is happening in intercultural interactions and mindful attention to cultural cues [6]. Items 2, 4, 7, 9, 12, 15, 18, 20 and 22 are reverse coded. The lowest total score that can be obtained from the scale is 24, and the highest score is 120. An increase in the score obtained from the scale indicates an increase in the level of intercultural sensitivity. The Cronbach’s alpha value of the scale was found to be 0.72. In the present study, the Cronbach’s alpha value of the scale was found to be 0.872.

### Data collection

The data of the present study were collected using an online survey method between May 8 and June 11, 2023, in the 2022–2023 Academic Year, following ethics committee and institutional approval. The survey link was distributed via WhatsApp class-group announcements and the official social media accounts of the relevant faculty; access to these channels was restricted to students enrolled in the department. On the first page of the online questionnaire, participants were presented with an information sheet describing the study aim and procedures, the voluntary nature of participation, and confidentiality/anonymity considerations, along with the research team’s contact details. Participants provided electronic informed consent (by selecting the “I agree to participate” option) before proceeding to the survey items, and they were informed that they could withdraw at any time by exiting the survey (e.g., closing the page) without any consequences.

### Data analysis

Data analysis was performed using IBM SPSS Statistics for Windows, Version 22.0. Categorical variables were summarized as frequencies and percentages, and continuous variables as means and standard deviations. The distribution of continuous variables was evaluated using skewness and kurtosis values and was considered acceptable within the commonly used ± 1.5 range; therefore, parametric tests were applied. Group comparisons were conducted using independent samples t-tests and one-way ANOVA, with Tukey’s post-hoc test for multiple comparisons. Effect sizes were reported as Cohen’s d (t-tests), eta-squared (η²) (ANOVA), and Pearson’s r (correlations) [18]. Multiple linear regression analysis was performed to identify independent predictors of xenophobia scores; age and grade were entered as continuous predictors, binary variables were entered as indicator-coded variables, and interaction frequency and geographic area were modeled using dummy variables (reference categories: daily interaction and southern group, respectively). Missing data were handled using listwise deletion. Multicollinearity was assessed using tolerance and variance inflation factor (VIF) values, and model assumptions were checked using standard residual diagnostics (ZRESID–ZPRED plot, histogram, and normal P–P plot); influential observations were examined via Cook’s distance and leverage values [19]. Statistical significance was set at *p* < 0.05.

### Ethical considerations

Approval was received from the Mardin Artuklu University Non-Interventional Ethics Committee (03.05.2023, 2023/5 − 4). Electronic informed consent was obtained from the participants with an electronic informed consent form, and the study was conducted in full compliance with the ethical principles of the Declaration of Helsinki [20].

## Results

Participants (*n* = 472) were nursing (45.3%) and midwifery (54.7%) students with a mean age of 21.34 ± 2.84 years (range: 17–45). Most participants were female (84.1%) and had spent most of their lives in the Southeastern Anatolia Region (88.1%). Nearly three-quarters reported a desire to live abroad (73.1%). More than one-third of the students reported communicating with people from other countries via social media (36.7%). In addition, 41.3% had previously cared for a patient from a culture different from their own, and 26.5% reported language-related communication difficulties during caregiving (Table 1).

**Table 1:** Sociodemographic characteristics of the students (*n* = 472)

(*n* = 472)	*n*	%
**Department**		
Nursing	214	45.3
Midwifery	258	54.7
**Age (years)**,** mean ± SD = 21.34 ± 2.84 (range 17–45)**		
17–21 years old	288	61.0
22 and above	184	39.0
**Sex**		
Female	397	84.1
Male	75	15.9
**Grade**		
1st grade	101	21.4
2nd grade	133	28.2
3rd grade	143	30.3
4th grade	95	20.1
**Region where most of life was spent**		
Marmara, Aegean, Mediterranean, Central Anatolia	27	5.7
Southeastern Anatolia	416	88.1
Eastern Anatolia	29	6.1
**Do you have friends outside your culture?**		
Yes	371	78.6
No	101	21.4
**Have you been to another country before?**		
Yes	53	11.2
No	419	88.8
**Do you have citizenship of another country?**		
Yes	33	7.0
No	439	93.0
**How often do you interact with people from different cultures/countries?**		
Every day	49	10.4
Once a week	48	10.2
Once a month	78	16.5
Less than once a month	212	44.9
Other	85	18.0
**Do you communicate with people from other countries over social media?**		
Yes	173	36.7
No	299	63.3
**Would you like to live abroad?**		
Yes	345	73.1
No	127	26.9
**Have you ever cared for a patient from a culture foreign to you?**		
Yes	195	41.3
No	277	58.7
**Have you experienced any problems during the caregiving process? If so**,** please briefly describe them.**		
Yes ***(language-related communication problem)***	125	26.5
No	347	73.5

The mean Xenophobia Scale score was 51.83 ± 12.74 (range: 11–66). The mean Intercultural Sensitivity Scale score was 88.25 ± 12.63 (range: 58–118). The subdimension means of the Intercultural Sensitivity Scale were 25.81 ± 4.58 for responsibility in interaction, 22.48 ± 3.87 for respect for cultural differences, 17.30 ± 3.87 for self-confidence in interaction, 11.20 ± 2.54 for enjoying the interaction, and 11.44 ± 2.26 for being careful in interaction (Table 2).

**Table 2:** Mean scores of students on the xenophobia and intercultural sensitivity scales (*n* = 472)

	Mean	SD	Min-Max
**Xenophobia Scale**	51.83	12.74	11–66
**Intercultural Sensitivity Scale**	88.25	12.63	58–118
Responsibility in Interaction	25.81	4.58	11–35
Respect for Cultural Differences	22.48	3.87	8–30
Self-confidence	17.30	3.87	5–25
Enjoying the Interaction	11.20	2.54	3–15
Be Careful in Interaction	11.44	2.26	3–15

Group comparisons are presented in Table 3. Xenophobia scores were higher among students aged ≥ 22 years than those aged 17–21 (t=-3.929, *p* < 0.001; d = 0.37) and differed by grade (F = 4.931, *p* = 0.002; η²=0.03; Tukey: 3rd/4th year > 1st year). Xenophobia also differed by the geographic area where students had spent most of their lives (F = 9.818, *p* < 0.001; η²=0.04; Tukey: Eastern and Southeastern Anatolia> Marmara/Aegean/Mediterranean/Central Anatolia). Markedly lower xenophobia was found among students who had been abroad (t=-8.120, *p* < 0.001; d = 1.18) and those holding citizenship of another country (t=-5.936, *p* < 0.001; d = 1.07).

**Table 3:** Differences in xenophobia and intercultural sensitivity scores according to demographic characteristics

(*n* = 472)	Xenophobia Scale Mean ± SD	Intercultural Sensitivity Scale Mean ± SD	Responsibility in Interaction Mean ± SD	Respect for Cultural Differences Mean ± SD	Self-confidence Mean ± SD	Enjoying the Interaction Mean ± SD	Be Careful in Interaction Mean ± SD
**Department**							
Nursing	51.55 ± 12.78	87.50 ± 12.00	25.36 ± 4.45	22.42 ± 3.94	17.20 ± 3.93	11.23 ± 2.51	11.27 ± 2.27
Midwifery	52.07 ± 12.72	88.86 ± 13.11	26.19 ± 4.66	22.53 ± 3.82	17.38 ± 3.83	11.17 ± 2.57	11.57 ± 2.25
t	-0.439	-1.161	-1.943	-0.308	-0.508	0.255	-1.442
p	0.66	0.24	0.053	0.75	0.61	0.79	0.15
Effect size (**Cohen’s d)**	0.04	0.11	**0.18**	0.03	0.05	0.02	0.13
**Age (Mean ± SD = 21.34 ± 2.84; min = 17-max = 45)**							
17–21 years old	50.02 ± 13.14	88.56 ± 13.00	25.88 ± 4.76	22.72 ± 3.89	17.26 ± 3.91	11.29 ± 2.53	11.38 ± 2.25
22 and above	54.67 ± 11.56	87.76 ± 12.04	25.71 ± 4.28	22.09 ± 3.83	17.36 ± 3.81	11.05 ± 2.55	11.52 ± 2.29
t p	-3.929* **0.00**	0.672 0.50	0.401 0.68	1.719 0.08	-0.264 0.79	0.994 0.32	-0.662 0.50
Effect size (**Cohen’s d)**	**0.37**	0.06	0.04	0.16	0.03	0.09	0.06
**Sex**							
Female	51.87 ± 12.50	88.28 ± 12.58	25.97 ± 4.57	22.56 ± 3.86	17.17 ± 3.86	11.07 ± 2.51	11.48 ± 2.25
Male	51.62 ± 14.03	88.05 ± 12.95	24.97 ± 4.57	22.01 ± 3.94	17.97 ± 3.86	11.90 ± 2.62	11.18 ± 2.31
t p	0.157 0.875	0.147 0.883	1.744 0.08	1.139 0.255	-1.632 0.103	-2.618* **0.009**	1.059 0.290
Effect size (**Cohen’s d)**	0.02	0.02	0.22	0.14	0.21	**0.33**	0.13
**Grade**							
1st grade	48.67 ± 13.59	90.93 ± 13.00	26.76 ± 4.91	23.38 ± 3.70	17.42 ± 4.28	11.65 ± 2.40	11.70 ± 2.20
2nd grade	50.48 ± 13.03	87.69 ± 13.19	25.57 ± 4.66	22.54 ± 4.28	17.02 ± 3.85	11.26 ± 2.62	11.29 ± 2.52
3rd grade	54.27 ± 12.53	88.81 ± 12.94	25.94 ± 4.65	22.26 ± 3.66	17.74 ± 3.95	11.25 ± 2.40	11.60 ± 2.14
4th grade	53.42 ± 10.79	85.32 ± 10.22	24.95 ± 3.78	21.75 ± 3.63	16.91 ± 3.26	10.57 ± 2.69	11.11 ± 2.09
F p Difference: Tukey	4.931* **0.002** **3.4 > 1**	3.444* **0.017** **1 > 4**	2.733* **0.043** **1 > 4**	3.135* **0.025** **1 > 4**	1.194 0.312	3.039* **0.029** **1 > 4**	1.556 0.199
Effect size (**η2: Eta-squared**)	**0.03**	**0.02**	**0.02**	**0.02**	0.01	**0.02**	0.01
**The geographic area spent most of life**							
Marmara, Aegean, Mediterranean, Central Anatolia	41.85 ± 18.50	90.77 ± 15.25	26.74 ± 4.69	22.85 ± 4.81	18.62 ± 3.98	11.74 ± 3.09	10.81 ± 2.78
Southeastern Anatolia	52.62 ± 11.81	88.07 ± 12.28	25.76 ± 4.48	22.43 ± 3.82	17.20 ± 3.87	11.20 ± 2.47	11.47 ± 2.16
Eastern Anatolia	49.79 ± 15.37	88.37 ± 14.93	25.68 ± 5.76	22.86 ± 3.73	17.48 ± 3.68	10.75 ± 3.00	11.58 ± 2.99
F p Difference: Tukey	9.818* **0.000** **2.3 > 1**	0.580 0.560	0.584 0.558	0.298 0.742	1.748 0.175	1.045 0.353	1.128 0.325
Effect size (**η2: Eta-squared**)	**0.04**	0.00	0.00	0.00	0.01	0.00	0.01
**(** ***n*** ** = 472)**	**Xenophobia Scale** **Mean ± SD**	**Intercultural Sensitivity Scale** **Mean ± SD**	**Responsibility in Interaction** **Mean ± SD**	**Respect for Cultural Differences** **Mean ± SD**	**Self-confidence** **Mean ± SD**	**Enjoying the Interaction** **Mean ± SD**	**Be Careful in Interaction** **Mean ± SD**
**Do you have friends outside your culture?**							
Yes No	51.87 ± 13.12 51.71 ± 11.29	89.12 ± 12.84 85.04 ± 11.31	26.05 ± 4.67 24.95 ± 4.11	22.57 ± 3.89 22.13 ± 3.81	17.63 ± 3.89 16.09 ± 3.57	11.35 ± 2.58 10.67 ± 2.33	11.50 ± 2.24 11.18 ± 2.34
t p	0.112 0.911	2.901* **0.004**	2.154* **0.032**	1.001 0.317	3.573* **0**,**000**	2.382* **0.018**	1.264 0.207
Effect size (**Cohen’s d)**	0.01	**0.33**	**0.24**	0.11	**0.40**	**0.27**	0.14
**Have you been to another country before?**							
Yes	39.28 ± 18.16	87.33 ± 14.38	25.15 ± 5.46	22.75 ± 3.88	18.05 ± 4.55	11.03 ± 2.90	10.33 ± 2.96
No	53.42 ± 10.92	88.36 ± 12.40	25.90 ± 4.45	22.44 ± 3.87	17.21 ± 3.77	11.22 ± 2.49	11.58 ± 2.12
t p	-8.120* **0.000**	− 0.557 0.578	-1.125 0.261	0.545 0.586	1.501 0.134	− 0.509 0.611	-3.808* **0.000**
Effect size (**Cohen’s d)**	**1.18**	0.08	0.16	0.08	0.22	0.07	**0.56**
**Do you have citizenship of another country?**							
Yes	39.57 ± 16.83	85.54 ± 18.27	25.30 ± 6.10	21.30 ± 4.90	17.90 ± 4.92	10.96 ± 2.86	10.06 ± 2.93
No	52.76 ± 11.90	88.45 ± 12.10	25.85 ± 4.45	22.56 ± 3.78	17.25 ± 3.78	11.22 ± 2.52	11.54 ± 2.17
t p	-5.936* **0.000**	-1.276 0.202	− 0.669 0.504	-1.814 0.070	0.929 0.354	− 0.551 0.582	-3.676* **0.000**
Effect size (**Cohen’s d)**	**1.07**	0.23	0.12	0.33	0.17	0.10	**0.66**
**How often do you interact with people from different cultures/countries?**							
Every day^1^	45.65 ± 17.86	89.83 ± 14.76	25.71 ± 5.16	22.44 ± 4.10	19.14 ± 4.36	11.32 ± 2.83	11.20 ± 2.64
Once a week ^2^	51.39 ± 14.73	91.04 ± 14.50	26.87 ± 4.76	21.89 ± 4.44	18.72 ± 4.12	11.56 ± 2.94	11.97 ± 2.30
Once a month ^3^	52.69 ± 12.55	87.57 ± 13.18	25.92 ± 4.79	22.26 ± 3.96	17.23 ± 3.36	11.16 ± 2.48	10.98 ± 2.21
Less than a month ^4^	54.34 ± 8.93	87.44 ± 12.14	25.46 ± 4.50	21.41 ± 3.75	16.98 ± 3.81	11.21 ± 2.35	11.37 ± 2.25
Other ^5^	51.83 ± 12.74	88.37 ± 10.70	26.07 ± 4.08	23.20 ± 3.60	16.31 ± 3.51	10.94 ± 2.67	11.84 ± 1.98
F P	3.916* **0.004**	1.051 0.380	1.040 0.386	1.081 0.365	6.420* **0**,**000**	0.496 0.738	2.344 0.068
Difference: Tukey	**1 < 3**,**4**,**5**				**1 < 3**,**4**,**5**		
Effect size (**η2: Eta-squared**)	**0.03**	0.01	0.01	0.01	**0.05**	0.00	**0.02**
**Do you communicate with people from other countries over social media?**							
Yes	50.59 ± 14.46	89.69 ± 13.55	26.20 ± 4.80	22.38 ± 4.13	18.28 ± 4.00	11.35 ± 2.51	11.46 ± 2.33
No	52.55 ± 11.59	87.41 ± 12.00	25.59 ± 4.43	22.53 ± 3.72	16.73 ± 3.68	11.12 ± 2.56	11.42 ± 2.22
t p	-1.615 0.107	1.894 0.059	1.388 0.166	− 0.399 0.690	4.274* **0.000**	0.955 0.340	0.158 0.874
Effect size (**Cohen’s d)**	0.15	0.18	0.13	0.04	**0.41**	0.09	0.02
**(** ***n*** ** = 472)**	**Xenophobia Scale** **Mean ± SD**	**Intercultural Sensitivity Scale** **Mean ± SD**	**Responsibility in Interaction** **Mean ± SD**	**Respect for Cultural Differences** **Mean ± SD**	**Self-confidence** **Mean ± SD**	**Enjoying the Interaction** **Mean ± SD**	**Be Careful in Interaction** **Mean ± SD**
**Would you like to live abroad?**							
Yes	53.45 ± 12.24	88.44 ± 12.89	25.86 ± 4.60	22.49 ± 3.95	17.42 ± 3.93	11.13 ± 2.50	11.51 ± 2.21
No	47.44 ± 13.07	87.73 ± 11.91	25.67 ± 4.54	22.44 ± 3.67	16.97 ± 3.68	11.39 ± 2.64	11.24 ± 2.39
t p	4.647* **0.000**	0.540 0.590	0.404 0.686	0.136 0.892	1.119 0.264	− 0.975 0.330	1.144 0.253
Effect size (**Cohen’s d)**	**0.48**	0.06	0.04	0.01	0.12	0.10	0.12
**Have you ever cared for a patient from a culture foreign to you?**							
Yes	53.31 ± 12.46	87.61 ± 13.35	25.69 ± 4.90	22.03 ± 4.03	17.34 ± 3.92	11.27 ± 2.54	11.27 ± 2.39
No	50.80 ± 12.85	88.69 ± 12.09	25.90 ± 4.34	22.79 ± 3.74	17.27 ± 3.84	11.15 ± 2.54	11.55 ± 2.16
t p	2.116* **0.035**	− 0.916 0.360	− 0.499 0.618	-2.124* **0.034**	0.205 0.838	0.474 0.635	-1.360 0.175
Effect size (**Cohen’s d)**	**0.20**	0.09	0.05	**0.20**	0.02	0.05	0.12
**Have you experienced any problems during the caregiving process? If yes**,** please briefly describe them.**							
Yes	52.47 ± 12.11	89.22 ± 12.03	26.45 ± 4.22	22.77 ± 3.99	17.58 ± 3.62	10.90 ± 2.69	11.50 ± 2.02
No	51.61 ± 12.97	87.89 ± 12.83	25.58 ± 4.68	22.37 ± 3.83	17.20 ± 3.96	11.31 ± 2.48	11.41 ± 2.34
t p	0.647 0.518	1.006 0.315	1.821 0.069	0.992 0.322	0.939 0.348	-1.547 0.123	0.364 0.716
Effect size (**Cohen’s d)**	0.07	0.11	0.19	0.10	0.10	0.16	0.04

t: Independent Sample T-Test; F: One-Way Analysis of Variance (ANOVA); Difference: Tukey *: *p* < 0.05

*d*: Cohen’s d (Effect size for t-test), η2: Eta-squared (Effect size for ANOVA)

Xenophobia varied by the frequency of intercultural interaction (F = 3.916, *p* = 0.004; η²=0.03; post-hoc differences are shown in Table 3) and was higher among students who expressed a desire to live abroad (t = 4.647, *p* < 0.001; d = 0.48). Students who had previously cared for a patient from a foreign culture had slightly higher xenophobia (t = 2.116, *p* = 0.035; d = 0.20) (Table 3).

Intercultural sensitivity total scores differed by grade (F = 3.444, *p* = 0.017; η²=0.02; Tukey: 1st year > 4th year) and were higher among students reporting friends outside their culture (*p* = 0.004) (Table 3). At the subdimension level, responsibility in interaction, respect for cultural differences, and enjoyment of interaction also differed by grade (all *p* < 0.05). Male students reported higher enjoyment of interaction than female students (t=-2.618, *p* = 0.009; d = 0.33).

Students communicating with people from other countries via social media reported higher self-confidence in interaction (t = 4.274, *p* < 0.001; d = 0.41), while those who had not been abroad or did not hold another citizenship scored higher on being careful/attentive in interaction (both *p* < 0.001; d = 0.56 and d = 0.66, respectively). Students who had cared for a patient from a foreign culture reported slightly lower respect for cultural differences (t=-2.124, *p* = 0.034; d = 0.20) (Table 3).

Pearson correlation analysis showed no significant association between total xenophobia and total intercultural sensitivity scores (*r* = 0.045, *p* = 0.326). Xenophobia was weakly and positively correlated with responsibility in interaction (*r* = 0.105, *p* = 0.023) and being careful in interaction (*r* = 0.243, *p* < 0.001) (Table 4).

**Table 4:** The relationship between students’ xenophobia scale and intercultural sensitivity scale scores

		Xenophobia Scale	Intercultural Sensitivity Scale	Responsibility in Interaction	Respect for Cultural Differences	Self-confidence	Enjoying the Interaction	Be Careful in Interaction
**Xenophobia Scale**	r	1.000						
	p							
**Intercultural Sensitivity Scale**	r	0.045	**1.000**					
	p	0.326						
Responsibility in Interaction	r	0.105*	0.864**	**1.000**				
	p	**0.023**	**0.000**					
Respect for Cultural Differences	r	-0.087	0.754**	0.613**	**1.000**			
	p	0.060	**0.000**	**0.000**				
Self-confidence	r	0.006	0.682**	0.455**	0.206**	**1.000**		
	p	0.889	**0.000**	**0.000**	**0.000**			
Enjoying the Interaction	r	-0.058	0.618**	0.317**	0.430**	0.427**	**1.000**	
	p	0.210	**0.000**	**0.000**	**0.000**	**0.000**		
Be Careful in Interaction	r	0.243**	0.677**	0.610**	0.417**	0.342**	0.216**	**1.000**
	p	**0.000**	**0.000**	**0.000**	**0.000**	**0.000**	**0.000**	

r: Pearson Correlation Coefficient *:*p* < 0.05 **:*p* < 0.01

### Additional analysis of the “desire to live abroad” variable

In the bivariate comparisons, students who reported a desire to live abroad had higher xenophobia scores than those who did not (Table 3). To examine whether this pattern could be explained by differences in other characteristics, the variable was also evaluated in the multivariable model. After adjustment for age, grade, prior caregiving for a foreign patient, geographic area (dummy-coded), and intercultural interaction frequency (dummy-coded; reference: daily interaction), desire to live abroad remained independently associated with higher xenophobia (B = 5.834, *p* < 0.001) (Table 5).

**Table 5:** Multiple linear regression model predicting xenophobia scale total score (*n* = 472)

Predictor	B	SE	β	t	*p*	95% CI (Lower, Upper)	Tolerance	VIF
Constant	37.465	5.200	—	7.205	**< 0.001**	27.246, 47.683	—	—
Age	0.148	0.213	0.033	0.694	0.488	−0.270, 0.565	0.825	1.212
Grade	1.510	0.632	0.123	2.389	**0.017**	0.268, 2.752	0.698	1.433
Cared for a foreign patient	−1.711	1.262	−0.066	−1.355	0.176	−4.191, 0.770	0.780	1.282
Interaction frequency (Once a week vs. Every day)	6.159	2.430	0.146	2.534	**0.012**	1.383, 10.935	0.559	1.790
Interaction frequency (Once a month vs. Every day)	6.628	2.191	0.193	3.025	**0.003**	2.322, 10.933	0.455	2.197
Interaction frequency (Less than a month vs. Every day)	6.792	1.933	0.265	3.513	**< 0.001**	2.993, 10.591	0.326	3.068
Interaction frequency (Other vs. Every day)	10.277	2.182	0.310	4.709	**< 0.001**	5.988, 14.566	0.429	2.333
Desire to live abroad (Yes vs. No)	5.834	1.257	0.203	4.639	**< 0.001**	3.363, 8.305	0.969	1.032
Marmara, Aegean, Mediterranean, Central Anatolia (vs. Southeastern Anatolia)	−9.430	2.413	−0.172	−3.908	**< 0.001**	−14.172, − 4.688	0.960	1.042
Eastern Anatolia (vs. Southeastern Anatolia)	−2.813	2.312	−0.053	−1.216	0.224	−7.357, 1.731	0.978	1.023

Model fit: R²=0.143; Adjusted R²=0.124; F(10,461) = 7.680; *p* < 0.001

B = Unstandardized Coefficient; SE = Standard Error; β = Standardized Coefficient; VIF = Variance Inflation Factor

Notes: Reference categories were daily interaction for intercultural interaction frequency and the southern group for geographic area. Dummy/indicator variables were coded as 1 = category present and 0 = otherwise

In the multiple linear regression analysis, the overall model was significant (R²=0.143; adjusted R²=0.124; F(10,461) = 7.680, *p* < 0.001). Using daily interaction with people from different cultures as the reference category, xenophobia scores were higher among students reporting interaction once a week (B = 6.159, *p* = 0.012), once a month (B = 6.628, *p* = 0.003), less than once a month (B = 6.792, *p* < 0.001), and those reporting other interaction patterns (B = 10.277, *p* < 0.001).

Students who wished to live abroad had higher xenophobia scores than those who did not (B = 5.834, *p* < 0.001). Grade level was also positively associated with xenophobia (B = 1.510, *p* = 0.017).

With respect to geographic area, the western group had lower xenophobia scores compared with the reference geographic group (Southeastern Anatolia) (B = − 9.430, *p* < 0.001), whereas the eastern group indicator was not significant (*p* = 0.224). Age and prior caregiving for a foreign patient were not significant predictors in the adjusted model (*p* > 0.05). Collinearity diagnostics indicated no evidence of problematic multicollinearity (VIF range: 1.03–3.07; tolerance range: 0.33–0.98). (Table 5).

## Discussion

This study examined xenophobia and intercultural sensitivity among nursing and midwifery students within the context of increasingly multicultural healthcare environments. The findings provide insight into how negative and positive orientations toward cultural diversity may coexist among future healthcare professionals. The following discussion interprets the main findings in relation to existing literature and considers possible contextual and educational influences associated with these attitudes.

The mean xenophobia score found in this study is consistent with previous studies conducted with nursing and midwifery students reporting moderate levels of xenophobic attitudes [10, 12, 21–24]. The presence of measurable xenophobic attitudes among future healthcare professionals is noteworthy, as such attitudes may influence communication with culturally diverse patients and the perceived quality of care. Although the present study did not directly assess clinical behaviour, moderate xenophobic attitudes among nursing and midwifery students may still have implications for culturally responsive care. Such attitudes may influence communication with culturally diverse patients, reduce sensitivity to cultural needs and preferences, and contribute to difficulties in establishing therapeutic relationships in multicultural healthcare settings [11, 12]. In addition, negative attitudes toward culturally different groups may limit openness to intercultural interaction and reduce willingness to engage in culturally responsive care practices [4, 13]. These findings highlight the potential importance of educational approaches aimed not only at improving intercultural communication skills but also at fostering reflective awareness and reducing exclusionary attitudes during professional training. Increasing cultural diversity in healthcare settings has been associated with the need for stronger intercultural competence among healthcare workers [4, 11], and previous studies have suggested that negative attitudes toward migrants may coexist with professional knowledge and skills [10, 12].

Age was associated with xenophobia scores, with students aged 22 years and older reporting higher scores than younger students. Previous research has reported inconsistent findings regarding age, with some studies showing no association and others reporting differences depending on context [7, 25]. Several contextual and sociocultural factors may help explain this finding. Older students may have had greater exposure to migration-related social discourse, negative media representations, or community-level tensions associated with cultural diversity over time [10]. In addition, prolonged exposure to sociopolitical debates related to migration and resource distribution may contribute to more negative attitudes toward culturally different groups, particularly in regions experiencing intense migration movements. Educational factors may also play a role. If intercultural and culturally responsive care content is not reinforced longitudinally throughout the curriculum, the potential positive effects of early educational exposure may diminish over time. However, because the present study was cross-sectional, it is not possible to determine whether age itself or accumulated social and educational experiences explain this difference.

Grade level was also associated with xenophobia, with higher scores found in upper-year students compared with first-year students. Similar findings have been reported in some nursing student samples [7, 12, 24], whereas other studies have found no difference or lower xenophobia in senior students [10, 25]. These inconsistent results suggest that progression through the curriculum alone does not necessarily appear to be associated with reduced negative attitudes, and that structured and continuous integration of intercultural content may be worth exploring in future curriculum development. Previous studies have emphasized that short or purely theoretical training may have limited impact on intercultural attitudes [26, 27].

Geographic background was another factor associated with xenophobia. Students who had spent most of their lives in the Marmara, Aegean, Mediterranean, or Central Anatolia regions reported lower xenophobia scores than those from Eastern and Southeastern Anatolia. Regional differences in socioeconomic conditions, migration patterns, and exposure to cultural diversity have been reported to influence attitudes toward migrants and minority groups [12, 26]. However, the present study did not directly measure these contextual factors, and these differences should therefore be interpreted cautiously.

Students who had been abroad or who held citizenship in another country reported substantially lower xenophobia scores. Previous studies have also suggested that international experience and intercultural contact are associated with more positive attitudes toward culturally different groups [11, 12, 14]. It is possible that direct exposure to different cultural environments is associated with lower uncertainty and perceived threat, although the cross-sectional design of the present study does not allow conclusions about the direction or nature of this relationship.In contrast, students who expressed a desire to live abroad had higher xenophobia scores, and this association remained significant in the regression analysis. Migration intention may reflect economic or educational expectations rather than attitudes toward immigration into one’s own society, and similar inconsistencies have been noted in previous research [23]. This seemingly paradoxical finding may reflect that migration intention is often motivated by economic or professional mobility rather than openness to immigration within one’s own society. In other words, students may perceive migration as an individual career opportunity while still holding reservations about immigration in their local social context. Future qualitative studies may help clarify whether this finding reflects economic mobility aspirations, dissatisfaction with local opportunities, or more complex perceptions regarding migration and cultural diversity.

The frequency of intercultural interaction was also related to xenophobia. Students who reported daily interaction with people from different cultures had lower xenophobia scores than those reporting less frequent contact. This finding is consistent with studies suggesting that social contact may be associated with lower prejudice [7, 10, 28]. However, the effect size was small, and the present study did not evaluate the quality of these interactions. Previous literature indicates that contact may reduce negative attitudes only under certain conditions, such as equal status and meaningful communication [14].

In the bivariate analysis, students who had previously cared for a patient from a culture different from their own showed slightly higher xenophobia scores; however, this variable was not a significant predictor in the adjusted regression model. Clinical encounters with culturally diverse patients may involve communication difficulties, uncertainty, emotional discomfort, or challenges related to unfamiliar cultural expectations, particularly when students feel insufficiently prepared for intercultural care [2, 29, 30]. In the present study, a considerable proportion of students also reported language-related communication problems during caregiving, which may have contributed to stressful or less effective intercultural clinical experiences. Inadequate preparation for culturally responsive care and limited opportunities for structured reflection during clinical practice may further influence how students interpret and respond to these encounters [13, 31]. These findings suggest that clinical exposure alone may not necessarily reduce xenophobic attitudes and that intercultural clinical experiences may be more beneficial when supported by structured educational content, communication training, and guided reflection.

Intercultural sensitivity scores in the present study were relatively high. These scores were similar to those reported in previous studies using the same scale [8, 22, 26]. Intercultural sensitivity differed by grade level, with first-year students scoring higher than fourth-year students. Previous studies have reported varying results, including higher sensitivity in early years, higher sensitivity in later years, or no difference [13, 26, 32]. These inconsistent findings suggest that multiple factors, including personal background, educational content, and clinical experiences may influence intercultural sensitivity.

Students who reported having friends from other cultures showed higher intercultural sensitivity, which is consistent with studies indicating that interpersonal contact is associated with greater cultural awareness [7, 26, 30]. Similarly, communication with people from other countries through social media was associated with higher self-confidence in intercultural interaction, in line with research suggesting that online intercultural communication may contribute to cultural learning [33]. These findings support the idea that both direct and indirect intercultural contact may be related to intercultural competence.

No significant correlation was found between total xenophobia and total intercultural sensitivity scores. However, weak positive correlations were found between xenophobia and the responsibility in interaction and being careful in interaction subdimensions. Previous studies have often reported an inverse relationship between intercultural sensitivity and xenophobia [7, 8, 12], but some research has also suggested that these constructs may not always move in opposite directions [9]. The present findings support the view that intercultural sensitivity and xenophobia may represent partly independent attitudinal dimensions, and that evaluating both constructs separately may provide a more accurate understanding of students’ readiness for culturally responsive care.

Taken together, these findings suggest that xenophobia and intercultural sensitivity among nursing and midwifery students may be shaped not only by individual characteristics but also by broader educational, sociocultural, and contextual influences.

These findings have implications at multiple levels. At the individual level, nursing and midwifery students may benefit from educational experiences that encourage reflective awareness of personal attitudes toward culturally diverse groups and strengthen intercultural communication skills. At the academic level, undergraduate curricula should move beyond solely knowledge-based approaches and incorporate structured, longitudinal educational strategies addressing both intercultural competence and xenophobic attitudes. Educational interventions such as simulation-based intercultural communication training, guided reflective practice, and supervised intercultural clinical experiences may support the development of culturally responsive care competencies. Training content may include structured intercultural communication skills, cultural humility, reflective practice, bias awareness, and guided discussions addressing stereotypes, discrimination, and equitable care in multicultural healthcare settings. Strengthening these competencies during undergraduate education may contribute not only to improved culturally responsive care practices but also to more inclusive professional relationships, greater patient trust, and reduced inequalities in healthcare delivery.

At the clinical practice level, healthcare institutions and clinical educators may play an important role in supporting students during intercultural patient encounters by facilitating communication support, mentorship, and reflective discussion opportunities. At the societal level, broader migration-related social discourse, media representations, and sociocultural tensions may also influence students’ attitudes toward culturally diverse groups, highlighting the importance of socially inclusive and equity-oriented educational approaches.

Future research should employ longitudinal, multi-center, and mixed-method designs to better understand how intercultural experiences, educational interventions, and clinical exposure influence the development of xenophobia and intercultural sensitivity over time.

### Limitations

This study has several limitations. First, the cross-sectional design precludes causal inferences regarding the relationships between xenophobia and intercultural sensitivity. Second, although the Xenophobia Scale and Intercultural Sensitivity Scale used in the study were previously validated instruments with established psychometric properties, some demographic and experiential variables were collected using a researcher-developed introductory information form. In addition, all data were collected through an online self-report survey, which may be subject to response bias and social desirability bias. Third, the study was conducted at a single state university, which may limit the generalizability of the findings to other educational or cultural contexts. Finally, the quantitative design may not fully capture the complexity and contextual nature of students’ attitudes toward cultural diversity. Future research using longitudinal, multi-center, and mixed-method designs is recommended to provide a more comprehensive understanding of these constructs.

## Conclusion

This study examined xenophobia and intercultural sensitivity among nursing and midwifery students and identified several associated sociodemographic and experiential factors. Although intercultural sensitivity levels were relatively high, moderate levels of xenophobic attitudes were also observed. The absence of a significant relationship between total xenophobia and intercultural sensitivity scores suggests that these constructs may function as partly independent attitudinal dimensions rather than opposite ends of a single continuum.

These findings suggest that nursing education may benefit from addressing not only intercultural communication skills but also underlying attitudes toward migrants and culturally diverse populations. Based on the present findings and the existing literature, incorporating structured and longitudinal educational strategies—such as simulation-based training, guided reflection, and meaningful intercultural contact—may be a promising direction for supporting the development of culturally responsive healthcare professionals. However, the effectiveness of such strategies requires further empirical evaluation. Future research using longitudinal and multi-institutional designs is recommended to further explore how educational experiences influence the development of these attitudes over time. Addressing xenophobia alongside intercultural sensitivity may represent an important step toward preparing future nurses and midwives for equitable and culturally responsive healthcare delivery.

## Data Availability

The datasets used and/or analyzed during the current study are available from the corresponding author on reasonable request.

## Declarations

### Generative AI and AI-assisted technologies in the manuscript preparation process

During the preparation of this work, the authors used ChatGPT, Gemini, and DeepL for language editing, grammar correction, translation and back-translation, and improvement of clarity. The authors reviewed and revised all outputs and take full responsibility for the final content of the manuscript. All citations, interpretations, and conclusions are the authors’ own.

## References

[1] Bilgiç Ş, Şahin İ. Intercultural sensitivity and ethnocentrism levels of nursing students. Süleyman Demirel Univ J Health Sci. 2019;10(3):230–6.

[2] Köse Tosunöz İ, Öztürk Çopur E. The relationship between ethnocentrism and xenophobia level and predictors: A descriptive and correlational study of nurses working in two cities where refugees live intensively in Turkey. Nurs Health Sci. 2024;26(1):e13107. 38566443 10.1111/nhs.13107

[3] Bozdağ F, Kocatürk M. Zenofobi Ölçeği’nin geliştirilmesi: Geçerlik ve güvenirlik çalışmaları. J Int Soc Res. 2017;10(52):615–20.

[4] Tanrıverdi G. Approaches and recommendations for improving the cultural competence in nursing. Florence Nightingale J Nurs. 2017;25(3):227–36.

[5] van der Veer K, Yakushko O, Ommundsen R, Higler L. Cross-national measure of fear-based xenophobia: Development of a cumulative scale. Psychol Rep. 2011;109(1):27–42. 22049645 10.2466/07.17.PR0.109.4.27-42

[6] Chen GM, Starosta WJ. The development and validation of the Intercultural Sensitivity Scale. Hum Commun. 2000;3:1–15.

[7] Dursun Ergezen F, Aydın R. The relationship between intercultural sensitivity, ethnocentrism, socio-demographic characteristics and xenophobia in nursing students: A descriptive and multi-centric study. Nurse Educ Today. 2025;144:106443. 39383659 10.1016/j.nedt.2024.106443

[8] Yıldız M, Yıldırım MS, Elkoca A, Varol E, Aydın MA, Dege G. Investigation of the relationship between xenophobic attitude and intercultural sensitivity level in health education students. J Transcult Nurs. 2023;34(3):238–46. 36927307 10.1177/10436596231158136

[9] Akca A, Ayaz-Alkaya S. Determinants of attitudes towards refugees and intercultural sensitivity of nursing students: A descriptive and correlational study. Nurse Educ Today. 2023;124:105772. 36889047 10.1016/j.nedt.2023.105772

[10] Mert-Karadas M, Kovanci MS, Ocalan S, Uslu-Sahan F, Ozdemir L. The tendency and affecting factors of nursing students’ xenophobia toward refugees from the perspective of ecological systems theory: A convergent parallel mixed method study. Nurse Educ Pract. 2024;78:104013. 38879910 10.1016/j.nepr.2024.104013

[11] White JA, Blaauw D, Rispel LC. Social exclusion and the perspectives of health care providers on migrants in Gauteng public health facilities, South Africa. PLoS ONE. 2020;15(12):e0244080. 33370340 10.1371/journal.pone.0244080 PMC7769451

[12] Güngör S, Tosun B, Prosen M. The relationship between intercultural effectiveness and intercultural awareness and xenophobia among undergraduate nursing and vocational schools of health services students: A descriptive study. Nurse Educ Today. 2021;107:105104. 34438306 10.1016/j.nedt.2021.105104

[13] Tosun B, Sinan Ö. Knowledge, attitudes and prejudices of nursing students about the provision of transcultural nursing care to refugees: A comparative descriptive study. Nurse Educ Today. 2020;85:104294. 31786486 10.1016/j.nedt.2019.104294

[14] Genkova P, Schreiber H. If you like one, you like them all? The relationship of intercultural competence, contact, and xenophobia. Int J Divers Educ. 2023;23(2):93–117.

[15] UNHCR. Türkiye fact sheet: February 2023 [Internet]. 2023 [cited 2026 Feb]. Available from: https://www.unhcr.org/tr/sites/tr/files/legacy-pdf/Bi-annual-fact-sheet-2023-02-Turkiye-.pdf

[16] Özmete E, Yıldırım H, Serdarhan D. Adaptation of the scale of xenophobia to Turkish culture: Validity and reliability study. J Soc Policy Stud. 2018;18(40/2):191–209.

[17] Bulduk S, Tosun H, Ardıç E. Türkçe kültürler arası duyarlılık ölçeğinin hemşirelik öğrencilerinde ölçümsel özellikleri. Turkiye Klinikleri J Med Ethics Law Hist. 2011;19(1):25–31.

[18] Lakens D. Calculating and reporting effect sizes to facilitate cumulative science: A practical primer for t-tests and ANOVAs. Front Psychol. 2013;4:863. 24324449 10.3389/fpsyg.2013.00863 PMC3840331

[19] Kim JH. Multicollinearity and misleading statistical results. Korean J Anesthesiol. 2019;72(6):558–69. 31304696 10.4097/kja.19087 PMC6900425

[20] World Medical Association. World Medical Association Declaration of Helsinki: ethical principles for medical research involving human subjects. JAMA. 2013;310(20):2191–4. 24141714 10.1001/jama.2013.281053

[21] Akçoban S, Şahmaran T. Sağlık teknikeri adaylarının kültürel farkındalık ve yabancı düşmanlığı düzeyleri: Meslek yüksekokulu örneği. Göbeklitepe Sağlık Bilimleri Dergisi. 2022;5(9):9–18.

[22] Durat G, Tarsuslu B. The relationships between nursing and midwifery students’ cultural sensitivity and xenophobia. Ege Univ Hemşirelik Fak Derg. 2022;38(1):11–20.

[23] Özdemir S, Sevinç S. Correlation between cultural competence, xenophobia, and attitudes toward brain drain in nursing students. Nurse Educ Today. 2023;131:105963. 37734367 10.1016/j.nedt.2023.105963

[24] Yekeler B, Şahin M. Göçmenlerin ülkemizdeki sağlık yüküne etkisi ve göçmenlere bakış açısı: Sağlık personeli aday örneği. Gümüşhane Univ J Health Sci. 2021;10(1):98–104.

[25] Demir E. Sağlık çalışanlarında kültürlerarası iletişim kaygısının zenofobi (yabancı düşmanlığı) ile ilişkisi [master’s thesis]. Gaziantep: Hasan Kalyoncu University. 2021.

[26] Güner S, Ocak Aktürk S, Öner Aydın S, Karaca Saydam B. Investigation of intercultural sensitivity and ethnocentrism levels of midwife candidates in Turkey sample: A cross-sectional study. J Transcult Nurs. 2022;33(2):208–18. 34865576 10.1177/10436596211057914

[27] Limoges J, Nielsen K, MacMaster L, Kontio R. Globally networked learning: Deepening Canadian and Danish nursing students’ understanding of nursing, culture and health. Nurse Educ Today. 2019;76:228–33. 30849667 10.1016/j.nedt.2019.02.006

[28] Bozdağ F. Xenophobia and social contact in university students. Int J Educ Lit Stud. 2020;8(4):87–97.

[29] Kaya Y, Arslan S, Erbaş A, Yaşar BN, Küçükkelepçe GE. The effect of ethnocentrism and moral sensitivity on intercultural sensitivity in nursing students: A descriptive cross-sectional study. Nurse Educ Today. 2021;100:104867. 33740704 10.1016/j.nedt.2021.104867

[30] Kılıç SP, Sevinç S. The relationship between cultural sensitivity and assertiveness in nursing students from Turkey. J Transcult Nurs. 2018;29(4):379–86. 28826358 10.1177/1043659617716518

[31] Karatay G, Bowers B, Karadağ EB, Demir MC. Cultural perceptions and clinical experiences of nursing students in eastern Turkey. Int Nurs Rev. 2016;63(4):547–54. 27682150 10.1111/inr.12321

[32] Baksi A, Sürücü HA, Duman M. Evaluation of the intercultural sensitivity and related factors of nursing students. J Acad Res Nurs. 2019;5(1):31–9.

[33] Carlson E, Stenberg M, Chan B, Ho S, Lai T, Wong A, et al. Nursing as universal and recognisable: Nursing students’ perceptions of learning outcomes from intercultural peer learning webinars: A qualitative study. Nurse Educ Today. 2017;57:54–9. 28732210 10.1016/j.nedt.2017.07.006

